# Telomeres susceptibility to environmental arsenic exposure: Shortening or lengthening?

**DOI:** 10.3389/fpubh.2022.1059248

**Published:** 2023-01-10

**Authors:** Kyi Mar Wai, Thinzar Swe, Maw Thoe Myar, Cindy Rahman Aisyah, Thae Su Su Hninn

**Affiliations:** ^1^Department of Social Medicine, Graduate School of Medicine, Hirosaki University, Hirosaki, Japan; ^2^Department of Human Ecology, Graduate School of Medicine, The University of Tokyo, Tokyo, Japan; ^3^Pre-clinical Department, University of Medicine 2, Yangon, Myanmar; ^4^Pre-clinical Department, University of Medicine Taunggyi, Taunggyi, Myanmar; ^5^Department of Geography, University of Yangon, Yangon, Myanmar

**Keywords:** telomere, telomerase, arsenic, metal, trace element (TE)

## Abstract

Maintaining telomere length plays a crucial role in regulating cellular life span. Telomere lengthening or shortening is one of the important biomarkers which could predict the preceding or present diseases. Meanwhile, the impact of environmental arsenic exposure on telomere length has increasingly concerned. Although previous studies demonstrated the effects of arsenic on telomere length, the findings were unclear on whether telomere shortens or lengthens by arsenic exposure. Thus, this manuscript summarized and discussed the telomere length alteration following arsenic exposure and the possible does-response effect of arsenic on telomere length. The present review suggested that different age groups may respond differently to arsenic exposure, and the dose-response effect of arsenic could be a critical factor in its effect on telomere length. Moreover, speciation analysis of arsenic could be more informative in identifying the effect of arsenic on telomere length.

## 1. Introduction

Telomeres are repetitive DNA oligomer sequences (TTAGGG) located at the ends of the eukaryotic chromosomes (1, 2). They prevent chromosomal damage by protecting against chromosome-chromosome fusions and degradation, thereby maintaining genomic integrity (1, 2). Telomere length is largely maintained by the telomerase RNA protein complex and the shelterin proteins, whose level is regulated by the telomerase reverse transcriptase (hTERT) and telomerase RNA (hTR) genes (1–5). Telomere shortening occurs in normal somatic cells following each cell division (1, 2). Once they reach a certain minimum length, cellular apoptosis occurs as a result of chromosomal end fusion (2). Telomerase can add the telomere DNA sequences (TTAGGG repeats) to prevent chromosomal fusion, and restore DNA lost to some extent (1–4). Dysfunctional telomeres and telomerase activity, in turn, may lead to genomic instability and malignancy (6–9). Therefore, maintaining optimal telomere length plays a crucial role in the regulation of cellular life span. Meanwhile, telomere length alteration was found to be correlated with aging and increased risks of many chronic diseases and malignancies (10–15).

Arsenic is a well-known hazardous metal naturally present in the environment and is also considered as a genotoxic metal (16, 17). In general, among two types of arsenic, organic and inorganic, inorganic arsenic is more toxic to humans (18). Natural contamination of inorganic arsenic in the groundwater is increasingly concerning for the health of residents in many countries where the groundwater is the primary source of arsenic exposure (19). Inorganic arsenic is exposed to human beings mainly through the oral route by drinking arsenic-contaminated water and food. Additionally, individuals can get exposed to arsenic *via* smoking either in passive or active ways (17–19). Globally, at least 200 million people in 140 countries have been threatened by arsenic toxicity acquired through drinking arsenic-contaminated water (18). Chronic arsenic exposure can result in significant health outcomes in the exposed population, including several diseases and cancer.

The impact of environmental and occupational heavy metals exposure on telomere length has increasingly concerned. Not an exclusion, arsenic exposure appears to modify the telomere length, which may predispose to adverse health risks (17, 20, 21). Arsenic induces oxidative stress and alters DNA repair and DNA methylation patterns, which could potentially affect telomere maintenance (16, 22–24). So far, many possible underlying mechanisms of arsenic-induced telomere length alteration have been proposed. Arsenic was shown to be involved in telomere shortening through the mediation of arsenic-induced apoptosis (21, 25). It is also possible that arsenic-induced oxidative stress directly damages the telomeres and telomere-related proteins (21, 26). The effect of arsenic on telomerase hTERT gene expression is also being concerned since telomerase activation plays a crucial role in telomere maintenance (26, 27).

Regarding the previous findings of arsenic-induced telomere length, the results are conflicting as some studies showed a positive association while others demonstrated a negative association between arsenic exposure and telomere length. These contrary results call to explore the knowledge gap on the effect of arsenic on telomere maintenance. Therefore, this manuscript summarized and discussed the telomere length alteration following arsenic exposure and the possible dose-response effect of arsenic on telomere length.

## 2. Mechanistic insight of arsenic-induced telomere length alteration

### 2.1. Arsenic-induced telomere lengthening

In the mechanistic insight, arsenic-induced telomere lengthening is caused by either telomerase-dependent or independent pathways. In the case of telomerase (hTERT) dependent pathway, the up-regulation of hTERT triggers telomere lengthening by synthesizing new telomere DNA sequences, and it was positively correlated with arsenic exposure (28–30). In accordance, a study conducted in Mongolia reported that hTERT expression was highly associated the arsenic exposure in both *in-vivo* and *in-vitro* studies (27). Moreover, hTERT expression is strongly linked to arsenic exposure despite its non-significant association with telomere length (31). Meanwhile, according to a case-control study in India, telomere lengthening could be telomerase-independent and triggered through the up-regulation of shelterin complex proteins such as TRF1 and TRF2, resulting in “alternative telomere lengthening (ATL)” (31). Alteration in these gene expressions may cause telomere de-protection, contributing to the activation of ATL (32).

A feasible telomerase-independent mechanism is the epigenetic modifications of subtelomeric DNA. In most cases, hypomethylation of subtelomeric DNA explains lengthening telomere, although some studies suggest hypermethylation in subtelomeric regions (33–36). A study in India revealed that chronic arsenic exposure lengthens telomere, and is correlated with subtelomeric methylation pattern changes (37). A couple of studies also demonstrated that telomere length is mainly altered by pattern changes in subtelomeric methylation, resulting in telomeric chromatin assemblage (35, 38). Thus, it is plausible for the distortion of telomere maintenance by the methylation pattern changes along with arsenic exposure and its effect on telomere length regulation.

### 2.2. Arsenic-induced telomere shortening

Arsenic-induced telomere shortening was well-known oxidative-stress-induced toxicity. Telomeres are made of high guanine content and are sensitive to oxidative stress; thus, high arsenic exposure may induce oxidative stress, rendering telomere attrition (21, 39–41). It was also reported that the primary mechanism behind the negative association between arsenic and telomere length was due to arsenic toxicity in the distortion of oxidative/anti-oxidant equilibrium (42, 43). This was probably facilitated by redox species overpowering anti-oxidant defenses, resulting in a shorter telomere, inflammation, and insufficient DNA repair mechanisms (17, 44–47).

Apart from the oxidative-stress-induced pathway, arsenic can trigger the fusion of chromosomal ends by downregulating telomere repeat binding factor in leukemia cell lines (26, 48). Some laboratory studies have also demonstrated that arsenic compound (As_2_O_3_) selectively suppresses the transcription of the hTERT gene by lowering the expression of some of the hTERT transcription factors in a dose-dependent manner (0.2-2 μM, 2.5 μM, 5 μM, and 10 μM, respectively) (29, 30). Further, arsenic can induce the up-regulation of heat shock proteins, oncogenes, and some cell growth factors that are also positive regulators of telomerase and cell proliferation (49).

## 3. Arsenic exposure and telomere length

### 3.1. Arsenic exposure and telomere length in adult populations

A couple of human population studies showed that the higher the arsenic exposure, the longer the telomere length (Table 1) (31, 32, 50, 52, 54, 55). For instance, a study conducted among Argentinian women supported the dose-related association (55). While the urinary arsenic levels of the participants varied from 0.1 to 1,251 μg/L, it is reported that an individual with an increased urinary arsenic tends to have longer telomeres (55). A case-control study, involving two groups of arsenic-exposed Indian adults—with skin lesions and without skin lesions in comparison with the control group, also revealed that the possible arsenic-induced lengthening was observed among the individuals with the skin-lesion (31). Likewise, 40 adults from India with neoplastic and surrounding normal tissues were recruited again 6 years later; not only was telomere length in tumor cells longer than that in normal cells, but higher arsenic levels also seemed to increase telomere length in that population (37). Based on the data from two large studies among the Bangladesh population, the same conclusion was drawn from 167 participants regarding the lengthening of telomeres in response to high arsenic concentration despite some controversial genetic results (32). The study in Mexico also casts light on arsenic′s role in lengthening telomere length (52). The study involved 188 Mexican males: 78 subjects with urine arsenic levels of 11.2–17.4 μg/L as an exposed group and 112 with lower concentrations as a control group (52). Similarly, in the study among Bolivians, the data from 193 recruited females reflected the correlation of arsenic exposure to telomere length as a biomarker of arsenic toxicity (50). The study also claimed that there is no gender difference in the associations between arsenic and telomere length (50).

**Table 1:** Summarized studies regarding the associations between arsenic exposure and telomere length in adults.

**Article**	**Study design**	**Study area/ sample size/ study population**	**Arsenic concentration (median or mean ±SD)**	**Telomere measurement method**	**Telomere length (TL) (median or mean ±SD)**	**Main finding**
De Loma et al. (50)	Cross-sectional	Bolivia, 193 females	Urine arsenic: 88 ± 69 μg/L; Blood arsenic: 2.4 ± 1.6 ng/g	qPCR	Relative TL: 1.0 ± 0.19	Higher urinary arsenic exposure is associated with longer TL.
Bhattacharjee et al. (37)	Cross-sectional	India, 40 subjects, each with tumor tissues and non-tumor controls	Urine arsenic: 218.86 ± 45.67 mg/L	qPCR	Relative TL: not mentioned (estimated range 1.1 ~ 3.3)	2-fold increase in exposure is associated with longer TL in 85% of arsenic-induced tumor tissues.
Srinivas et al. (51)	Case-control	Hungary, 528, Basal cell carcinoma patients, 533 healthy controls	Urine arsenic: 1.32 μg/L (both groups)	qPCR	Relative TL: 0.58 in patients, 0.40 in controls	Higher arsenic exposure (>1.32 μg/L) is associated with shorter TL.
Jimenez Villarreal et al. (52)	Case-control	Mexico, 188 males: 76 exposed and 112 controls	Urine arsenic: 497.5 ± 148.9 μg/mL/g creatinine in exposed group, and 151.1 ± 65.2 μg/mL/g in control group	qPCR	Relative TL: 0.71 ± 0.07 in exposed group, 0.54 ± 0.01 in control.	Longer TL in exposed group.
Zhang et al. (53)	Case control	Bangladesh, 1,469 subjects (Randomly selected sub-cohort)	Urine arsenic: 196 microgram/g	qPCR	Relative TL: 0.8	No association between baseline exposure and baseline TL.
Grau-Perez et al. (54)	Cohort	America, 1,702 Strong Heart Study (SHS) and 1,793 Strong Heart Family Study (SHFS) subjects	Urine arsenic: 8.8 μg/L (ΣAs) in SHS and 4.3 μg/L (ΣAs) in SHFS.	qPCR	Relative TL: 1.09 for SHS and 1.01 for SHFS	Higher exposure is associated with shorter TL in SHS but no association is found in SHFS.
Borghini et al. (42)	Cross sectional	Italy, 241 young adults	Urine total arsenic groups at ≥19.3 and ≥ 15 μg/L and inorganic arsenic at ≥ 3.86 μg/L	qPCR	Relative TL: not mentioned	Higher exposure is associated with shorter TL.
Gao et al. (32)	Cross sectional	Bangladesh, 1,966 adults from two studies	Urine arsenic: 336.7 μg/L ± 437.7	qPCR	Relative TL: 0.62	Higher exposure is associated with longer TL.
Chatterjee et al. (31)	Case-control	India, 180 individuals in West Bengal, India [60 with skin lesion (WSL), 60 without skin lesion (WOSL), and 60 controls]	Drinking water in μg/L: 153 ± 65.5 (WSL), 143 ± 59 (WOSL), 9.20 ± 0.31 (control); Urine arsenic in μg/L: 290 ± 83.4 (WSL), 318 ± 95.7 (WOSL), 30.5 ± 0.51 (control)	qPCR	Relative TL: 0.899 ± 0.084 (WSL), 0.767 ± 0.0728 (WOSL), 0.749 ± 0.044 (control)	Longer TL in WSL.
Li et al. (55)	Cross-sectional	Northern Argentina, 202 women	Urine arsenic: 230 μg/L	qPCR	Relative TL: 0.37	High urinary arsenic exposure is associated with longer TL.

qPCR, quantitative polymerase chain reaction; SD, standard deviation.

Meanwhile, there are some inconsistencies while the speciation analysis of arsenic is considered. In a large population study of the American Indian tribe, higher arsenic exposure tends to shorten telomere length among men and women (54). The average level of arsenic, calculated as the sum of inorganic and metabolite forms, was 8.8 μg/g creatinine. Whilst the negative association was more robust in the over-50 group, it was negligible among the middle-aged group (54). Another report investigating young male Italians from four arsenic-polluted regions also stated that long-term exposure to arsenic was associated with telomere attrition through DNA damage, resulting in shorter telomere length (42). The study evaluated the effects of arsenic metabolites on telomere length rather than the effect of total arsenic exposure. A case-control study with a lower arsenic exposure claimed a negative association between arsenic and telomere length (51). In addition, some studies reported no significant association between arsenic exposure and telomere length (53, 54). In summary, the previous literature among the human adult populations is conflicting in demonstrating the effect of arsenic on telomere length.

### 3.2. Early life arsenic exposure and telomere length in children and adolescents

Although the positive relationship was mainly observed in the adult population studies, there are some conflicting results in other age groups (Table 2). For example, from the study of 476 Bangladesh primary schoolers, urinary arsenic exposure (mean = 143.85 μg/L) was negatively associated with leucocyte telomere length (43). Specifically, with a 2-fold increase in urinary arsenic, telomere length was shortened by 0.017 relatively (43). Meanwhile, the results among childhood or adolescent populations are inconsistent. For instance, arsenic exposure was significantly and positively associated with telomere elongation in Mexican children (57). On the other hand, some studies showed no significant association between arsenic and telomere length as in Nepal and Indian populations (62, 63). In terms of exposure dose, Mexican elementary children had a mean urinary arsenic level of 54.78 μg/L, while the Indian and Nepal adolescents were in the higher range of 74.7 ± 34.9 μg/L and 114.5 ± 241.3 μg/L, respectively (62, 63). The exposure dose was lower than that of the previous study among Bangladesh children of similar age (43).

**Table 2:** Summarized studies regarding the associations between arsenic exposure and telomere length in children or adolescents.

**Article**	**Study design**	**Study area/ sample size/ study population**	**Arsenic concentration (median or mean ±SD)**	**Telomere measurement method**	**Telomere length (TL) (median or mean ±SD)**	**Main finding**
Smith et al. (56)	Prospective prebirth cohort	United States, 893 mothers and 408 newborns	Maternal blood arsenic: 1.3 ± 1.8 ng/g	qPCR	Relative TL: 0.64 ± 1.10 (maternal TL); 1.06 ± 0.48 (cord blood TL)	2-fold increase in maternal arsenic exposure is marginally associated with longer cord blood TL but not statistically significant.
Farzan et al. (43)	Cohort	Bangladesh, 476 mother-child pairs	Urine arsenic: 143.85 ± 157.57 μg/L	Novel Luminex Assay	Relative TL: 237 ± 49.8 (males); 239 ± 50.2 (females)	High urinary arsenic exposure is associated with shorter TL.
Alegría-Torres et al. (57)	Cross-sectional	Mexico, 88 children (6–15 years old)	Urine arsenic: 54.78 μg/L ± 32.59 μg/L	qPCR	Relative TL: 1.03 ± 0.74	Higher urinary arsenic exposure is associated with longer TL.
Song et al. (58)	Birth cohort	China, 762 mother-child pairs	Urine arsenic: 21.7 μg/g creatinine (1^st^ trimester), 27.3 μg/g creatinine (2^nd^ trimester), 27.1 μg/g creatinine (3^rd^ trimester)	qPCR	Relative TL: 0.75 (geometric mean)	2-fold increase in maternal arsenic exposure during the 3^rd^ trimester is associated with 5.75% increase in cord blood TL.
Herlin et al. (59)	Birth cohort	Northern Argentina, 194 pregnant women	24 μ g/L in maternal urine, 2.2 μg/L in maternal blood/ serum, 8.4 μg/L in placenta, 2.3 μg/L in core blood/ serum	qPCR	Relative TL: 1.26 ± 0.12 (cord blood); 0.77 ± 0.21 (placenta); 0.99 ± 0.31 (maternal blood in late pregnancy)	Maternal arsenic exposure is associated with longer telomere by 0.2 SD for each doubling.
Mannan et al. (60)	Birth cohort	Bangladesh, 4,436 mothers, 640 children (at 4.5 years) and 564 children (at 9 years)	Urine arsenic: mother−88 (median) (range 1.9–1576 μg/L), 4.5-year child-57.1 μg/L, 9-year child-53.9 μg/L	qPCR	Relative TL: 188.9 kb/dg (68.6–464.0) for 4.5 years; 164.8 kb/dg (53.3–455.0) for 9 years	Prenatal and childhood arsenic exposure is negatively associated with TL when urinary arsenic is higher than 45 μg/L, and positively associated with TL when it is lower than 45 μg/L.
Wai et al. (61)	Birth cohort	Myanmar, 409 mother-newborn pairs	Urine arsenic: 73.9 μg/g creatinine	qPCR	Relative TL: 0.9	High urinary arsenic exposure is associated with shorter TL.
Chatterjee et al. (62)	Case-control	India, 68 children (exposed), 52 children (control)	Urine arsenic: 74.7 ± 34.9 μg/L (exposed), 25.4 ± 4.86 μg/L (control)	qPCR	Relative TL: 0.85	TL in the arsenic exposed children was slightly higher than the unexposed. Not significant result.
Fillman et al. (63)	Cross-sectional	Nepal, 351 adolescents (12–16 years)	Urine arsenic: 74.7 ± 34.9 μg/L	qPCR	Relative TL: 1.69 ± 1.10	High urinary arsenic exposure is associated with longer TL, but not significant.

qPCR, quantitative polymerase chain reaction; SD, standard deviation.

In concern of the trans-placental transfer of arsenic, a couple of birth-cohort studies have been performed to examine the effect of prenatal arsenic exposure on the offspring's telomere length. A previous study in Wuhan (China) with 762 mother-newborn pairs reported a strong association between maternal arsenic exposure during the third trimester and the telomere length of the newborns (58). If arsenic exposure levels are raised by 2-fold among mature pregnant women, telomere length in their offspring is predicted to be longer by 5.75% (58). However, with blood arsenic levels being in the range of 1.3 ± 1.8 ng/g, there was only a marginal association in a similar prebirth cohort in the United States (56). Furthermore, a research team led by Herlin M, et al. (59) from Northern Argentina conducted a mother-child cohort study to pinpoint the consequences of multiple toxic metals including arsenic in the body, showing that the arsenic content in maternal blood and cord blood has no association with the offspring′s telomere length; arsenic concentration in the placenta blood was somehow found to have an association with telomere elongation in the offspring.

In contrast, a previous study in Myanmar among 409 mother-child pairs indicated a negative correlation between the telomere length in newborns and prenatal arsenic exposure (median urinary arsenic = 73.9 μg/g creatinine) (61). In another cohort study in Bangladesh, children from mothers with a higher arsenic concentration in urine or a higher arsenic exposed children had shorter telomeres when their urinary arsenic values exceeded 45 μg/L, demonstrating a negative relationship. Numerically, if the arsenic exposure was doubled, 34 kb/dg of telomere length was shortened. Interestingly, at below 45 μg/L, arsenic was found to be related to telomere elongation among the 9-year-olds, demonstrating a positive relationship (60). These contrary results suggest a call to explore more for an understanding dose-response effect of arsenic on telomere maintenance.

## 4. Considerations for the effects of arsenic on telomere length

### 4.1. A possible dose-response effect of arsenic on telomere length

Telomere susceptibility to arsenic exposure could be dose-dependent, and it would be worth focusing on the understanding of the dose-response effect of arsenic on telomere and telomerase. In the previous *in-vitro* study on human cord blood cells, it was stated that telomere length was lengthened at an inorganic arsenic exposure level of both 0.0001 μM and 1 μM after 24 h of treatment, while telomere length was shortened at a high concentration of 1 μM after 7 days in culture (28). Consistently, another study on human cell lines *in-vitro* also supported that telomerase activity induced by arsenite (AsIII) concentration at 0.1–1 μM resulted in telomere elongation, whereas a high concentration of more than 1–40 μM shortened telomere length (25). The above-mentioned findings highlight that it is necessary to investigate at which dose arsenic may activate the ALT pathway that leads to telomere lengthening or may induce oxidative pathway that leads to telomere shortening in the human population.

### 4.2. Differences in the responses by age groups

Most studies agreed upon the shortening effect of arsenic on telomere length among childhood and adolescents. Contrarily, a previous study among Mexican elementary children reported a telomere lengthening where the study population had a mean urinary arsenic concentration of 54.78 μg/L (57). In fact, the study among elementary Bangladesh children stated the opposite findings; their mean urinary arsenic level (143.8 μg/L) was twice that of the Mexicans′ study (42). The relationship among adolescents was non-significant according to Indians, whose concentration was at 74.7 μg/L (62). Nonetheless, telomere length was significantly shorter among high arsenic-exposed Nepali adolescents (114.5 μg/L) (63). According to these controversial relationships among childhood communities, it could be deduced that the larger exposure to arsenic could lead to telomere shortening while the smaller exposure might respond differently among children or adolescents.

The findings of some cohort studies that studied mother-newborn or mother-children's pairs are also controversial; two studies reckoned the association between arsenic and telomere length to be positive (58, 59); however, the two other papers revealed a negative association (60, 61). A study in Chinese mother-newborn pairs claimed a direct correlation between prenatal arsenic exposure, especially during the third trimester of pregnancy, and telomere length at a mean urinary arsenic concentration of 27.1 μg/g creatinine (58). In contrast, another Argentinian study claimed that telomere attrition was found only with higher urine arsenic content (mean = 124 μg/L) (59). Similarly, the Myanmar population with a slightly lower concentration on average at 73.9 μg/g creatinine showed a negative association between prenatal arsenic exposure and newborn telomere length (61). All the above three studies were further strengthened by the fourth birth cohort study in Bangladesh, which agreed on the dose-related interrelation, demonstrating a positive association at a level below the mean urine arsenic concentration of 45 μg/L and a negative association if above 45 μg/L (60).

On the other hand, most studies among the adult population somehow agreed with the results of telomere elongation by arsenic exposure. Although the mean or median value of exposure concentration varies widely across the populations, the positive association between arsenic exposure and telomere length was concluded in most of the studies. For example, higher arsenic exposure was associated with longer telomere length in the Indian population at a mean urinary arsenic level of 290 μg/L (31) and the South African population at a similar exposure dose (median = 230 μg/L) (55). A longer telomere was associated with very high exposure to arsenic (mean = 497.5 μg/L) in Mexican males (52). Meanwhile, the results are consistent in Bangladesh adults, where the mean arsenic concentration was about half of the above studies at 188 μg/L (32). In Bolivian women, the exposure dose was as low as 88 μg/L (50). Even though most exposure values are higher than those in younger populations, it is noteworthy that arsenic-induced telomere lengthening is prevalent in adulthood, thereby calling for further research on other possible contributing factors such as genetic polymorphism, occupation, living environment, and other stresses.

### 4.3. Differences in the responses by arsenic metabolites

Another point to be considered is the toxicity of the metabolites of arsenic. Even among inorganic forms of arsenic, arsenite (AsIII) is considered more toxic than arsenate (AsV), which is methylated by a specific enzyme, arsenite methyltransferase (AS3MT), to form end products monomethylarsinic acid (MMA), and dimethylarsinic acid (DMA) (50, 64). The relative fractions of MMA and DMA, which are produced as a result of a series of reduction and methylation reactions of inorganic arsenic, represent the effectiveness of arsenic metabolism (65). Since MMA and DMA are less toxic to tissues than AsIII or AsV, and are more readily excreted in the urine, the methylation process is thought to be a detoxification process (66). Meanwhile, higher urinary MMA levels imply more harmful to the body, promoting more arsenic-induced health conditions (66), whilst higher urinary DMA levels mean healthier, with a lower inner arsenic pool (64). Henceforth, while inorganic urinary arsenic contributes to telomere elongation, DMA has the opposite effect, according to Li′s study (55). Apart from these two end products, other metabolites, MMAIII and DMAIII are also released from the methylation process (64). Some *in-vitro* studies have reported that MMAIII is more reactive than AsIII and AsV and could result in DNA damage (23, 64).

Since the toxicity of arsenic varies by its metabolites, the efficiency of arsenic metabolism may alter the effect of arsenic on telomere length. In accordance, a previous study in Bolivia shows that the individuals with less efficient arsenic metabolism capacity showed a longer telomere length (50). Moreover, a study in Argentina argues that the positive association between arsenic and telomere length is more robust in individuals with less efficient arsenic metabolism (55). In addition, AS3MT, the most important metabolizing enzyme, could be of variants that could influence metabolic efficiency and vulnerability to arsenic toxicity (67–70). A previous study supports the fact by suggesting that people with the *AS3MT* haplotype 1 may have been linked to a slower and more hazardous metabolism of arsenic, resulting in a longer telomere than those without this haplotype (55). The study also revealed a positive trend between telomere length and arsenic exposure with increasing copy number of *AS3MT* haplotype 1 (55). Accordingly, previous studies also reported that the efficacy of arsenic metabolism is strongly and consistently predicted by genetic differences in AS3MT (68–71). Thus, the toxic effects of arsenic metabolites may have influenced the extent of cellular damage.

## 5. Conclusion

**Figure 1:**
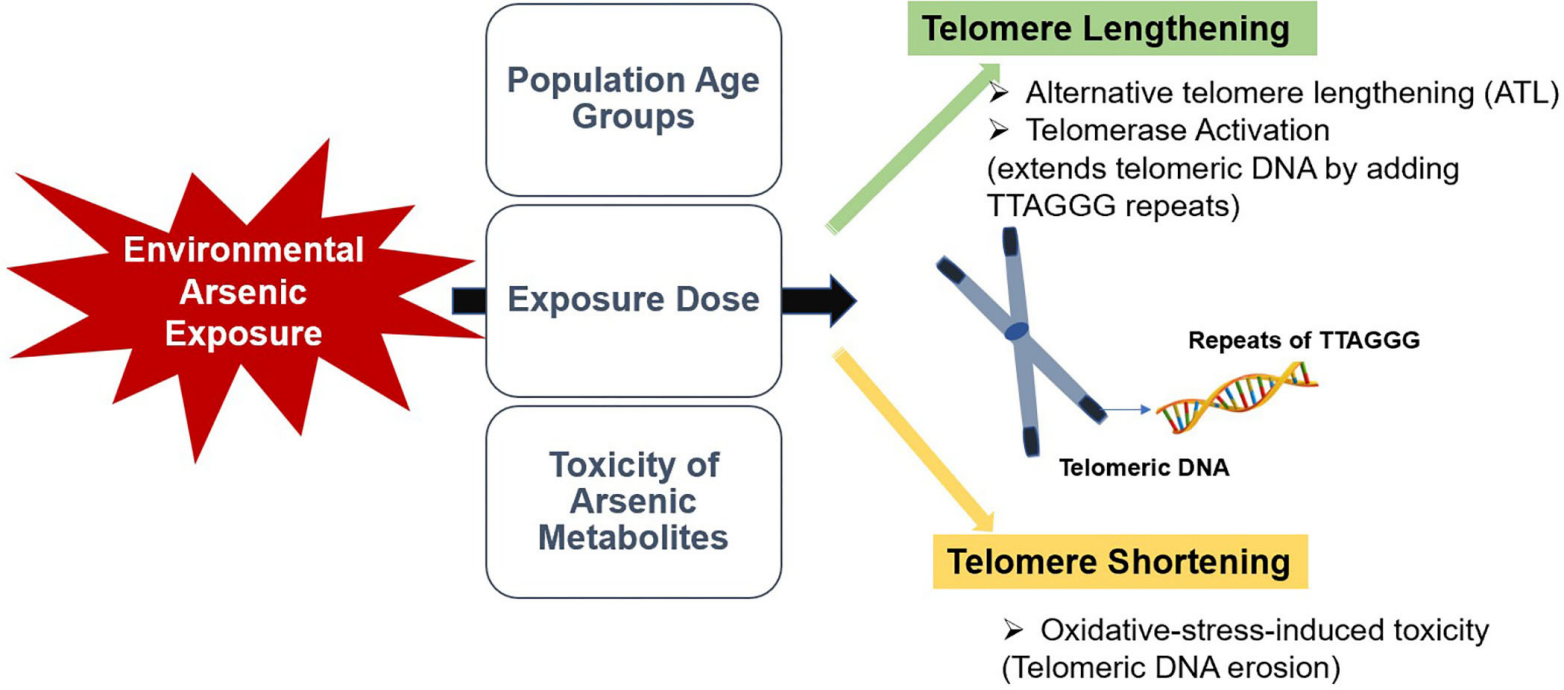
A summarized illustration of the study.

In conclusion, available information on the effects of environmental arsenic exposure on telomere length among human populations are controversial and limited. As summarized in Figure 1, this review suggested that different age groups may respond differently to arsenic exposure, and the dose-response effect of arsenic could be a critical factor in its effect on telomere length. Moreover, speciation analysis of arsenic could be more informative in identifying the effect of arsenic on telomere length. Targeting telomere and telomerase could be a possible rationale for alternative therapeutic and preventative strategies for age-related and malignant diseases. Consequently, assessment of telomere length in the early stage could be helpful in biomonitoring of health risks in later life. Therefore, further studies should focus on the dose-response effect of arsenic exposure on telomeres in human populations. Additionally, compelling comparative studies should be performed to determine the predictive impact of the toxicity of arsenic metabolites on telomere length to constitute a promising knowledge for early clinical and biological effects.

## Author contributions

KW designed and conceptualized the study. KW, TS, MM, and TH drafted the original manuscript. All authors critically reviewed, edited, and approved the manuscript.
